# The Association Between Age at First Live Birth and Depression: Results From NHANES 2005–2018

**DOI:** 10.1155/da/6614889

**Published:** 2025-09-30

**Authors:** Ali Kolahdooz, Fatemeh Movahed, Mohsen Yousefi, Amirhossein Salehi, Saba Goodarzi, Arman Shafiee

**Affiliations:** ^1^Student Research Committee, School of Medicine, Alborz University of Medical Sciences, Karaj, Iran; ^2^Non-Communicable Diseases Research Center, Alborz University of Medical Sciences, Karaj, Iran; ^3^Department of Gynecology and Obstetrics, Tehran University of Medical Sciences, Tehran, Iran; ^4^Student Research Committee, School of Medicine, Shahid Beheshti University of Medical Sciences, Tehran, Iran

**Keywords:** depression, depressive disorder, pregnancy

## Abstract

**Background:**

This cross-sectional study, utilizing National Health and Nutritional Examination Surveys (NHANESs) data from 2005 to 2018, examines the association between age at first live birth and depression among women aged 12 years or older.

**Methods:**

Data encompassed 10,399 participants, with 1260 exhibiting depressive symptoms. The 9-item Patient Health Questionnaire (PHQ-9) assessed depression. Age at first live birth was categorized as <18, 18–25, and >25.

**Results:**

Women with depressive symptoms were more likely to be single, have lower incomes and education levels, be smokers, and exhibit higher body mass indexes (BMIs) or sleep disorders. Younger age at first live birth correlated with higher depression prevalence. Univariate analysis shows decreased depression chances for women with first live births at 18–25 (47% decrease) or >25 (76% decrease), with an 11% reduction for every year increase in age at first birth. Multivariate analyses confirm a significant association between age at first live birth and depression, even after adjusting for various factors.

**Conclusion:**

This study underscores the association between age at first live birth and depression, highlighting the need for considering reproductive history in mental health assessments. The findings emphasize the multifaceted nature of this relationship, demonstrating the impact of sociodemographic and lifestyle factors on mental health outcomes among women.

## 1. Introduction

Depression, one of the most globally witnessed psychological health complications, can be the cause of lack of functionality, unhappiness, dissatisfaction, reduction in life quality of an individual, and a number of other concerning challenges [1]. Pregnancy is a period of time in which multiple factors can magnify the risk of depression; factors such as neuroendocrinological and psychosocial changes [2]. In a research conducted in Spain, the prevalence of major depression among 569 women ranging in age from 18 to 45 years was 5.1% in the first trimester, 4.0% in the second, and 4.7% in the third trimester [3]. Depressive symptoms not only occur during pregnancy but also are common after delivery, which gives us the term postpartum depression or PPD. While in developed countries the prevalence of PPD is from 5% to 30%, in developing countries the range is from 6.5% to 12.9% [4]. PPD can cause developmental impairments in new infants and in scarce occasions, infanticide or suicide among mothers [5].

As the World Health Organization declares, the average age at first birth is more than 25 among women worldwide, and the trend has an upward slope [6]. It has been long presumed that the lower the age at first birth is, the more possible it is to see psychiatric problems in a woman. Low-income women who give birth to their first offspring before the age of 19 are at higher stakes of developing psychiatric disorders and should be the target of intrapartum screening of anxiety disorders, including PTSD, and behavior disorders [7].

Postpartum depression and major depression disorder during pregnancy are two notice worthy psychiatric complications that pregnant women might go thorough perinatally. It has been assumed that lower ages at first birth can be an exposing factor for depression. There have been a few studies showing a possible correlation between lower ages at first labor and heightened chances of developing depression. For instance, it was found that there is a curvilinear relationship between mother's age and depression [8]. Moreover, another study found that there is a negative linear correlation between age at first birth and postpartum depression [9]. It was reported that the pivotal age splitting negative from positive correlation between age at first pregnancy and depressive disorders is nearly 23 in the USA [10].

Herein, we conducted a cross-sectional study, employing available data from the National Health and Nutritional Examination Surveys (NHANESs) to find a meaningful association between age at first birth and depression, both intrapartum and postpartum.

## 2. Methods

### 2.1. Data Source and Participants

This cross-sectional study analyzed publicly available data from the NHANESs. We focused on data collected between 2005 and 2018 for women aged 12 years or older, encompassing 10,399 participants. Of these, 1260 individuals reported symptoms of depression. The National Center for Health Statistics Research Ethics Review Board granted ethical approval for NHANES data usage.

### 2.2. Evaluation of Depressive Symptoms

Depressive symptoms were assessed using the 9-item Patient Health Questionnaire (PHQ-9), a tool based on the Diagnostic and Statistical Manual of Mental Disorders, Fourth Edition (DSM-IV) criteria for depression. Each question is scored on a scale from 0 (not at all) to 3 (nearly every day), yielding a total score between 0 and 27.

### 2.3. Assessment of Age at First Live Birth

Data on the age at first live birth was extracted from the NHANES Reproductive Health file, which includes questions on menstrual history, pregnancy history, hormone replacement therapy, and related topics. Female participants aged 12 and older were eligible to respond to question RHD180, which asked for their age at the time of their first live birth. Responses were categorized into three groups: under 18 years, 18–24 years, and 25 years or older.

### 2.4. Assessment of Covariates

NHANES interviewers collected data on a range of sociodemographic, lifestyle, and health factors during the in-home interview phase. Variables assessed included age, gender, ethnicity, education level, marital status, income-to-poverty ratio (where values below 1 indicate income below the poverty threshold), social support, physical activity, housing stability, smoking habits, and body mass index (BMI, measured in kg/m^2^). BMI was further categorized into underweight (<18.5), healthy weight (18.5–24.9), and overweight/obese (≥25). Alcohol consumption was defined as having at least 12 alcoholic drinks per year.

### 2.5. Statistical Analysis

Descriptive statistics were used to summarize participant demographics. Comparisons between participants with and without depression (PHQ-9 scores ≥10) were conducted using independent *t*-tests and design-based Pearson chi-square tests with Rao and Scott corrections to account for NHANES' complex survey design. The relationship between age at first live birth and depression was analyzed both linearly and categorically using univariate and multivariate logistic regression models. Marginal predictive plots illustrated the adjusted association between age at first birth and depression. Multivariate analyses controlled for potential confounders, including age, gender, race, BMI, alcohol consumption, smoking status, education (less than 12 years vs. more), income (above vs. below poverty level), and marital status (partnered vs. single). NHANES-recommended adjustments for primary sampling units, weights, and strata were incorporated into all analyses. Statistical significance was set at *p*  < 0.05, and analyses were performed using Stata version 16 (StataCorp LLC, Texas, USA).

## 3. Results

Table 1 displays the participant characteristics according to their present status of depression. Compared to women without depressive symptoms, women with current depressive symptoms were more likely to be single, have lower incomes, lower education levels, and be smokers. They were also more likely to have higher BMIs or sleep disorders. Compared to women who had their first kid at age 18–25 or beyond 25, those who had their first child under the age of 18 had a higher prevalence of depression (Table 2).

**Figure 1:**
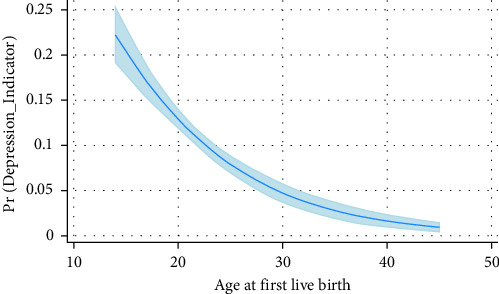
Marginal predictive plot based on the univariate regression model. The vertical coordinates represent the predicted probabilities of the dependent variable based on the model used in the analysis.

Table 3 shows the findings of the univariate analysis. Compared to women who gave birth before the age of 18, those who gave birth for the first time between the ages of 18 and 25 had a 47% decreased chance of developing depression, and those who gave birth for the first time at age 25 or later had a 76% decreased chance of developing depression. There was an 11% reduction in the likelihood of experiencing depression for every year of age at first birth (Figure 1).

**Figure 2:**
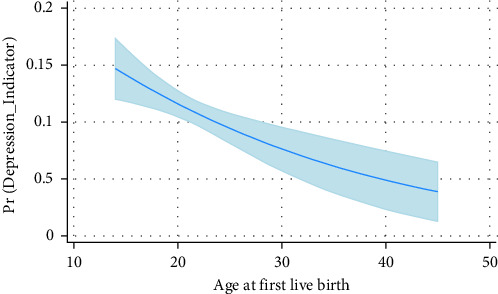
Marginal predictive plot based on the multivariate regression model. The vertical coordinates represent the predicted probabilities of the dependent variable based on the model used in the analysis.

Table 4 displays the results of multivariate analysis for the association between age at first live birth and depression (categorical outcome). Even after controlling for other variables, including marital status, income, education level, and sleep disorders, there was still a significant association between the age at first birth and depression. Women who gave birth between the ages of 18 and 25 and 25 and over had a 25% and 49% lower likelihood of developing depression than women who gave birth before the age of 18 (Figure 2).

## 4. Discussion

The purpose of the study was to look into the relationship between mother's age and depression both during and after pregnancy.

According to our findings, the prevalence of depression was higher among women who gave birth to their first child before becoming 18 than women who did so between the ages of 18 and 25 or beyond. The risk of developing depression was 47% lower in women who gave birth for the first time between the ages of 18 and 25 and 76% lower in women who gave birth for the first time at age 25 or more. For every year of life after the first birth, the chance of developing depression decreased by 11%. Even after multivariate analysis was used to compensate for other variables, this association remained valid.

Many studies investigating the association between depression and the age at first childbirth have shown that mother's age at first childbirth is linked to poor mental health [7, 10–14]. Aitken et al. [12] investigated the possible correlation between young maternal age at first birth and upcoming long-term mental health issues in women. They put the mental health of women who had their first child aged 15–19, 20–24, and 25 years and older into comparison using the mental health component summary score of the SF-36. Results indicated that there was an association between teenage births and poor mental health in teenage mothers [12].

Numerous studies have also found that a young mother's age at her first delivery is linked to postpartum depression [15–18]. In a cross-sectional study designed by Bottino et al. [17], among all participants, 197 were diagnosed with postpartum depression. An obvious association between maternal age and the occurrence of PPD was witnessed. This association was reportedly independent of socioeconomic and reproductive characteristics, conjugal status, or substance consumption by the parents. Furthermore, for each extra year delaying the pregnancy, a 4% reduction in the likelihood of developing postpartum depression could be foreseen [17]. In another study, Ou et al. [9] carried out a two-sample Mendelian randomization analysis and came into the conclusion that there is a causal effect between age at first birth and incidence of postpartum depression, meaning increased age at first birth decreases the chances of postpartum depression [9].

A study by Carlson [8] presented the findings of a multivariate analysis on a number of variables related to depression. In particular, a higher risk of depression is linked to an older age at first live birth, sleep disorders, non-Hispanic black and Mexican-American ethnicity, less education, being unmarried, having a lower income, smoking, and lack of physical activity. Furthermore, Muraca and Joseph [19] looked at the association between Canadian women's depression and age. The research discovered that, even after adjusting for other variables, including income, education, and marital status, a lower mother's age at her first childbirth was linked to a higher risk of depression. In particular, moms who gave birth between the ages of 20 and 34 were less likely to have depression than mothers who gave birth before the age of 20 [19]. Aasheim et al. [20] looked into the association between psychological distress in primiparous women from early pregnancy to 18 months postpartum and advanced maternal age. In total, 1507 primiparous women were enrolled in the study, which had a longitudinal design, from two public hospitals in Melbourne, Australia. In pregnancy and for up to 18 months after giving birth, higher levels of psychological distress were linked to older mothers, according to the study. In particular, the study discovered that women who were 35 years of age or older were more likely to have mental disorders than those who were 25–34 years old [20].

On the other hand, a prospective cohort study conducted by McMahon et al. [21] aimed to establish an association between early postpartum depression and the age group and method of conception of mothers. The individuals had assessments both during and 6 weeks after giving birth. According to the study, there is no evidence of a significant correlation between early postpartum depression and the age group or method of conception of the mother [21].

To summarize, numerous researches have consistently demonstrated that a woman's earlier age at her first live delivery is linked to a higher risk of developing depression. By using multivariate analysis to show that this link holds true even after adjusting for other variables, our study contributes to the body of evidence in this area.

Our study had numerous benefits, including the addition of socioeconomic status and habits, the use of a validated instrument to measure the outcome variable, and a large sample size. Our study does have several limitations, though. The cross-sectional design makes it more difficult for us to prove causation. Second, the true prevalence of depression may not be fully reflected in self-reported data due to recall bias. Furthermore, other variables, including genetic predisposition and social support, were not considered. In addition, it is proven that pregnant and breastfeeding women are susceptible to hormonal influences that can lead to inaccurate depression questionnaire scores [22]. However, the eligibility criteria for the depression screening questionnaire did not specify whether it included pregnant or breastfeeding women.

In conclusion, our research offers proof of the association between a woman's age at her first live birth and depression. These results emphasize the significance of early detection and treatment of depression symptoms in women who are or may become pregnant. In order to reduce the risk of depression in this population, future research should concentrate on identifying the underlying mechanisms and creating therapies. Early identification, mental health support, and community-based initiatives for young mothers should be given top priority in public health interventions. These results emphasize how important it is to take into account a woman's age at her first childbearing as a potential risk factor for depression. Note that this study does not establish a cause-and-effect relationship; rather, it just demonstrates an association between depression and the age at first birth. To ascertain the direction of the association and find any relevant confounding variables, more investigation is required.

## Figures and Tables

**Table 1:** Characteristics of participants by current depressive symptoms.

Variable	Depressed	No depression	Total (%)	*p*-Value
Age, mean (SE)	46.11 (0.49)	47.31 (0.29)		*p* < 0.001
Race (%)				*p* < 0.001
1. NHwhite	8.907	91.09	100	
2. NHblack	11.51	88.49	100	
3. MexAm	10.86	89.14	100	
4. Other	9.619	90.38	100	
Education (%)				*p* < 0.001
1. Less than 12 years	16.62	83.38	100	
2. More than 12 years	8.50	91.49	100	
Marital status (%)				*p* < 0.001
1. Single	12.85	87.15	100	
2. Married	7.572	92.43	100	
Income (%)				*p* < 0.001
1. Below poverty	19.26	80.74	100	
2. Above poverty	7.82	92.17	100	
Alcohol consumption (%)				*p*=0.054
1. Yes	9.907	90.09	100	
2. No	8.28	91.17	100	
Smoking (%)				*p* < 0.001
1. Yes	14.31	85.69	100	
2. No	6.81	93.19	100	
BMI (%)				*p* < 0.001
1. Normal	6.557	93.44	100	
2. Underweight	12.05	87.95	100	
3. Overweight	8.35	91.64	100	
4. Obese	12.83	87.17	100	
Sleep disorder (%)				*p* < 0.001
1. Yes	23.37	76.36	100	
2. No	6.119	93.88	100	

**Table 2:** Characteristics of participants by current depressive symptoms and age at first live birth.

Age group	Depression *(n* = 1260)	No depression (*n* = 9139)	Total (*n* = 10,399)	*p*-Value
Age at first birth (%)				
<18 years	20.05 (*n* = 329)	79.95 (*n* = 1323)	100 (*n* = 1652)	
18–25 years	11.73 (*n* = 738)	88.27 (*n* = 5230)	100 (*n* = 5968)	*p* < 0.001
>25 years	5.84 (*n* = 193)	94.16 (*n* = 2586)	100 (*n* = 2779)	

**Table 3:** Unadjusted odds ratios for variables associated with depression.

Variable	Unadjusted OR	CI 95%	*p*-Value
Age	0.9960	0.9926, 0.9947	*p*=0.02
Race			
1. NHwhite	Ref		
2. NHblack	1.3297	1.14, 1.54	*p* < 0.001
3. MexAM	1.2460	1.06, 1.45	*p* < 0.05
Education			
1. Below diploma	Ref		*p* < 0.001
2. Above diploma	0.4667	0.40, 0.53	
Marital status			
1. Single	Ref		*p* < 0.001
2. Married	0.5557	0.49, 0.62	
Income			
1. Below poverty	Ref		*p* < 0.001
2. Above poverty	0.3560	0.31, 0.40	
BMI			
1. Normal	Ref		
2. Underweight	1.9517	1.15, 3.29	*p*=0.013
3. Over weight	1.2998	1.09, 1.54	*p*=0.004
4. Obese	2.0973	1.76, 2.49	*p* < 0.001
Smoking			
1. No	Ref		*p* < 0.001
2. Yes	2.2835	1.98, 2.62	
Alcohol consumption			
1. No	Ref		*p*=0.055
2. Yes	1.1356	0.99, 1.29	
Sleep disorder			
1. No	Ref		*p* < 0.001
2. Yes	4.6796	4.12, 5.31	
Age at live birth (continuous)	0.8964	0.87, 0.91	*p* < 0.001
Age at live birth			
1. <18 years	Ref		
2. 18–25 years	0.5302	0.42, 0.66	*p* < 0.001
3. >25 years	0.2473	0.19, 0.32	*p* < 0.001

**Table 4:** Adjusted odds ratios for the association between age at first live birth and depression.

Age	Adjusted OR	CI 95%	*p*-Value
Age at first birth			
1. <18 years	Ref		
2. 18–25 years	0.7561	0.57, 0.99	*p*=0.044
3. >25 years	0.5142	0.35, 0.73	*p* < 0.001
Age at first birth (continuous)	0.9498	0.92, 0.97	*p*=0.001

## Data Availability

The data that support the findings of this study are available from the corresponding author upon reasonable request.

## References

[1] Aktas S., Yesilcicek Calik K. (2015). Factors Affecting Depression During Pregnancy and the Correlation Between Social Support and Pregnancy Depression. *Iranian Red Crescent Medical Journal*.

[2] Faisal-Cury A., Rossi Menezes P. (2007). Prevalence of Anxiety and Depression During Pregnancy in a Private Setting Sample. *Archives of Women’s Mental Health*.

[3] Míguez M. C., Vázquez M. B. (2021). Prevalence of Depression During Pregnancy in Spanish Women: Trajectory and Risk Factors in Each Trimester. *International Journal of Environmental Research and Public Health*.

[4] Howard L. M., Molyneaux E., Dennis C.-L., Rochat T., Stein A., Milgrom J. (2014). Non-Psychotic Mental Disorders in the Perinatal Period. *The Lancet*.

[5] Stein A., Pearson R. M., Goodman S. H. (2014). Effects of Perinatal Mental Disorders on the Fetus and Child. *The Lancet*.

[6] Balbo N., Billari F. C., Mills M. (2013). Fertility in Advanced Societies: A Review of Research. *European Journal of Population*.

[7] Tabet M., Flick L. H., Cook C. A., Xian H., Chang J. J. (2016). Age at First Birth and Psychiatric Disorders in Low-Income Pregnant Women. *Journal of Women’s Health*.

[8] Carlson D. L. (2011). Explaining the Curvilinear Relationship Between Age at First Birth and Depression Among Women. *Social Science & Medicine*.

[9] Ou Z., Gao Z., Wang Q., Lin Y., Ye D. (2023). Association Between Age at First Birth and Postpartum Depression: A Two-Sample Mendelian Randomization Analysis. *Heliyon*.

[10] Mirowsky J., Ross C. E. (2002). Depression, Parenthood, and Age at First Birth. *Social Science & Medicine*.

[11] Zasloff E., Schytt E., Waldenström U. (2007). First Time Mothers’ Pregnancy and Birth Experiences Varying by Age. *Acta Obstetricia et Gynecologica Scandinavica*.

[12] Aitken Z., Hewitt B., Keogh L., LaMontagne A. D., Bentley R., Kavanagh A. M. (2016). Young Maternal Age at First Birth and Mental Health Later in Life: Does the Association Vary by Birth Cohort?. *Social Science & Medicine*.

[13] Merikangas A. K., Calkins M. E., Bilker W. B., Moore T. M., Gur R. C., Gur R. E. (2017). Parental Age and Offspring Psychopathology in the Philadelphia Neurodevelopmental Cohort. *Journal of the American Academy of Child & Adolescent Psychiatry*.

[14] Fulco C. J., Henry K. L., Rickard K. M., Yuma P. J. (2020). Time-Varying Outcomes Associated With Maternal Age at First Birth. *Journal of Child and Family Studies*.

[15] Ghaedrahmati M., Kazemi A., Kheirabadi G., Ebrahimi A., Bahrami M. (2017). Postpartum Depression Risk Factors: A Narrative Review. *Journal of Education and Health Promotion*.

[16] Silverman M. E., Reichenberg A., Savitz D. A. (2017). The Risk Factors for Postpartum Depression: A Population-Based Study. *Depression and Anxiety*.

[17] Bottino M. N., Nadanovsky P., Moraes C. L., Reichenheim M. E., Lobato G. (2012). Reappraising the Relationship Between Maternal Age and Postpartum Depression According to the Evolutionary Theory: Empirical Evidence From a Survey in Primary Health Services. *Journal of Affective Disorders*.

[18] Peñacoba Puente C., Suso-Ribera C., Blanco Rico S., Marín D., San Román Montero J., Catalá P. (2021). Is the Association Between Postpartum Depression and Early Maternal–Infant Relationships Contextually Determined by Avoidant Coping in the Mother?. *International Journal of Environmental Research and Public Health*.

[19] Muraca G. M., Joseph K. S. (2014). The Association Between Maternal Age and Depression. *Journal of Obstetrics and Gynaecology Canada*.

[20] Aasheim V., Waldenström U., Hjelmstedt A., Rasmussen S., Pettersson H., Schytt E. (2012). Associations Between Advanced Maternal Age and Psychological Distress in Primiparous Women, From Early Pregnancy to 18 Months Postpartum. *BJOG: An International Journal of Obstetrics & Gynaecology*.

[21] McMahon C. A., Boivin J., Gibson F. (2011). Age at First Birth, Mode of Conception and Psychological Wellbeing in Pregnancy: Findings From the Parental Age and Transition to Parenthood Australia (PATPA) Study. *Human Reproduction*.

[22] Fairlie T. G., Gillman M. W., Rich-Edwards J. (2009). High Pregnancy-Related Anxiety and Prenatal Depressive Symptoms as Predictors of Intention to Breastfeed and Breastfeeding Initiation. *Journal of Women’s Health*.

